# Gene Expression Analysis of Biological Systems Driving an Organotypic Model of Endometrial Carcinogenesis and Chemoprevention

**DOI:** 10.4137/grsb.s344

**Published:** 2008-02-10

**Authors:** Doris M. Benbrook, Stan Lightfoot, James Ranger-Moore, Tongzu Liu, Shylet Chengedza, William L. Berry, Igor Dozmorov

**Affiliations:** 1 Department of Obstetrics and Gynecology, University of Oklahoma Health Sciences Center, Oklahoma City, OK; 2 Department of Biochemistry and Molecular Biology, University of Oklahoma Health Sciences Center, Oklahoma City, OK; 3 Department of Pathology, University of Oklahoma Health Sciences Center, Oklahoma City, OK; 4 Arizona Cancer Center, AZ; 5 Department of Cell Biology, University of Oklahoma Health Sciences Center, Oklahoma City, OK; 6 Oklahoma Medical Research Foundation, Oklahoma City, OK

**Keywords:** carcinogenesis, heteroarotinoid, chemoprevention, endometrial cancer, microarrays, karyometric analysis

## Abstract

An organotypic model of endometrial carcinogenesis and chemoprevention was developed in which normal endometrial organotypic cultures exposed to the carcinogen, DMBA (7,12-dimethylbenz[a]anthracene), developed a cancerous phenotype in the absence, but not presence of subsequent treatment with a flexible heteroarotinoid (Flex-Het), called SHetA2. A discriminant function based on karyometric features of cellular nuclei and an agar clonogenic assay confirmed these histologic changes. Interpretation of microarray data using an internal standard approach identified major pathways associated with carcinogenesis and chemoprevention governed by c-myc, p53, TNFα and Jun genes. Cluster analysis of functional associations of hypervariable genes demonstrated that carcinogenesis is accompanied by a stimulating association between a module of genes that includes tumor necrosis factor α (TNFα), c-myc, and epidermal growth factor-receptor (EGF-R) and a module that includes insulin-like growth factor I-receptor (IGF-IR), p53, and Jun genes. Two secreted proteins involved in these systems, tenascin C and inhibin A, were validated at the protein level. Tenascin C is an EGF-R ligand, and therefore may contribute to the increased EGF-R involvement in carcinogenesis. The known roles of the identified molecular systems in DMBA and endometrial carcinogenesis and chemoprevention supports the validity of this model and the potential clinical utility of SHetA2 in chemoprevention.

## Introduction

In comparison to classical approaches evaluating single molecules or pathways, a systems biology approach provides a broader perspective with the possibility of uncovering novel results in complex tissues, such as cancer of the uterine endometrium. The human endometrium consists of a complex mixture of cell types and extracellular matrix that is in a continual state of flux after puberty and before menopause. Communication between epithelial cells, stromal cells, and extracellular matrix is fundamental to endometrial cycling and function (Inaba et al. 1988; Bulletti et al. 1998). Furthermore, the ability of the epithelial and stromal cells to communicate through secretion of hormones and growth factors is affected by their proximity to each other (Hopfer et al. 1994). This communication is required for their coordination and production of extracellular matrix. Therefore, *in vitro* studies of endometrium should not rely solely on uniform cell lines grown in monolayers, but instead, should take into consideration the complex interaction of the different cell types within their extracellular matrix. Three-dimensional representations of tissue microenvironment can be provided by organotypic cultures consisting of cells grown in extracellular matrix materials to mimic tissue (Benbrook, 2006). To develop a model system that incorporated the complexity of the endometrial microenvironment, we cultured primary human endometrial cells inside and on top of collagen gels in filter inserts and demonstrated that hormonal treatments of these cultures induced tissue architecture reflective of different phases of the menstrual cycle (Kamelle et al. 2002). Both single stromal cells and epithelial glands developed inside the collagen gels, which is in contrast to a more recently developed model that separated the epithelial and stromal cells by culturing the epithelial cells as organoids inside matrigel-filled filter inserts and the stromal cells as monolayers on the plastic below the inserts (Bläuer et al. 2005).

The objective of this study was to further develop our organotypic model to study endometrial carcinogenesis and chemoprevention from a systems biology perspective that captured the complexity of tissue communication. Endometrial cancer is the most common female pelvic malignancy, but it is relatively understudied in comparison to other cancers. Tens of thousands of hysterectomies are performed every year for endometrial cancer and its preneoplastic lesions. Women with high risk factors for endometrial cancer, including patients with hereditary nonpolyposis colorectal cancer syndrome (HNPCC) (Broaddus et al. 2006), could benefit from diagnostic tests to identify early stage cancers and non-toxic chemoprevention agents.

Retinoids represent a promising class of chemoprevention agents that are modeled after retinoic acid, but are limited by their toxicity (Collins et al. 1999; Benbrook, 2002; Silverman et al. 1987). We developed a class of synthetic retinoids, called heteroarotinoids (Hets), that exhibit reduced toxicity in comparison to retinoic acid, but only moderate growth inhibition of cancer cell lines (Benbrook et al. 1998; Dhar et al. 1999; Zacheis et al. 1999). Our second generation Hets, called Flexible Hets or Flex-Hets, were modified to have increased flexibility in their chemical structure. These compounds are similar to retinoids in that they induce G1 cell cycle arrest and differentiation, but differ from conventional retinoids in that they are potent inducers of apoptosis and do not activate retinoid nuclear receptors (Guruswamy et al. 2001; Liu et al. 2004; Benbrook et al. 2005). The induction of apoptosis is much greater in cancer cells over normal cells and occurs through direct effects on mitochondria (Liu et al. 2007a). In our studies and those of collaborators, SHetA2 induced the highest levels of apoptosis and differentiation of all retinoids tested (Guruswamy et al. 2001; Chun et al. 2003; Liu et al. 2004). This lead compound also inhibited growth of OVCAR-3 ovarian and Caki renal tumor xenografts without evidence of toxicity (Benbrook et al. 2005). The lack of retinoid receptor activation by SHetA2 is supported by evidence of its lack of teratogenicity and topical irritancy in animal models (Benbrook et al. 2005; Mic et al. 2003). Because of these encouraging results, SHetA2 was chosen for preclinical development as a cancer therapeutic agent in the National Cancer Institute’s Rapid Access to Intervention Development (RAID) program (Application 196, Compound NSC 726189) and now is being developed as a cancer chemopreventive agent in the Rapid Access to Preventive Intervention Development (RAPID) program.

The molecular mechanism of SHetA2 induction of apoptosis in cancer cells is associated with decreases in Bcl-2 and Bcl-xl levels (Liu et al. 2007a), while G1 cell cycle arrest is associated with decrease of Cyclin D1 levels (Masamha, 2007; Liu et al. 2007b) and induction of differentiation is associated with increase of E-Cadherin expression (Liu et al. 2007b). The initiating event for these effects may be due to glutathione depletion caused by covalent binding of SHetA2 to glutathione (Chengedza et al. 2007; Liu et al. 2007c). Glutathione levels are important for maintaining mitochondrial and cellular redox status. The glutathione depletion by SHetA2 leads to inhibition of the activity of a redox-regulated transcription factor that regulates the aforementioned genes, nuclear factor—κB (NF-κB) (Chengedza et al. 2007; Liu et al. 2007c).

The complex response of gene expression patterns in endometrial organotypic cultures treated with carcinogens and potential chemoprevention agents, such as SHetA2, can be modeled with systems biology. Microarrays represent a powerful tool to generate hypothesis about the molecular mechanisms driving biological systems. The tremendous amount of information that can be obtained from microarray studies however, presents many challenges for data analysis including high levels of inaccuracy caused by the mutually exclusive characteristics of sensitivity and specificity. While statistical methods based on conventional t tests provide the probability (P) that a difference in gene expression occurred by chance, conventional thresholds for statistical tests of biological systems produce thousands of false positive selections in microarray experiments containing hundreds of thousands measurements. The straightforward approach to decrease these false positives by using high numbers of replicates is very expensive and inefficient. Correction of the P threshold by dividing the desired significance by the total number of statistical tests performed (Bonferroni correction), ensures achievement of a desired false-positive rate over the entire set of genes, but arguably sets a criterion to be too strict for each individual gene. Thus, the problem of specificity is solved at the consequence of decreasing the sensitivity. The most popular attempt to improve the Bonferroni adjustment is the False Discovery Rate (FDR) control in multiple hypotheses testing (Holm, 1979) introduced into microarray analysis by Benjamini and Hochberg (Benjamini et al. 1995). Instead of controlling for the chances for type I error across the entire set of hypotheses considered, FDR estimates and controls for the proportion of null hypotheses that are rejected. In the FDR method, all genes are ranked by their P values and tested against individualized thresholds: the smallest observed P value—against the strictest threshold, and the remaining P values against successively more relaxed thresholds. The use of individualized thresholds, as in the popular Significance Analysis of Microarrays (SAM) method (Tusher et al. 2001), improves the conservativeness of the Bonferroni test, although the improvement is only partial and often minor. This again results in a dependence on unrelated measurements, because the threshold adjustment for any given gene depends on the arbitrary number of genes with lower P values.

Fortunately, the power of statistical tests can be increased by taking advantage of the enormous quantities of information obtained in each microarray experiments. We developed a statistical approach of normalization and analysis procedures of microarray data that increases statistical power by using internal standards that characterize some aspect of system behavior, such as technical variability (Dozmorov et al. 2003a; Knowlton et al. 2004). In this internal standard approach, the paired comparison of gene expression in two different situations is accompanied by the associative test—checking the hypothesis that each given gene in the experimental group has common features and can be associated with an internal standard. The internal standard in this context is a large family of genes that share some features useful for analysis, which in turn have no dependence on the particular gene sequence, level of expression, or coordinates on the microarray chip used. For example, genes expressed below technical sensitivity comprise a background cohort (Dozmorov et al. 2004a), while genes exhibiting a similar expression pattern across several distinct experimental conditions are denoted as an equally expressed cohort. These internal standards are used to robustly estimate parameters that describe some state of the experimental system, such as the identification of genes expressed distinctly above background, differentially expressed genes, and genes having similar dynamical behavior. A direct comparison with the Bonferroni correction demonstrated advantages of the internal standard approach in that it produced selections with minimal false positive and false negative contaminations and discriminated between genes that are differentially expressed from those that are expressed only in one state. (Dozmorov et al. 2003a).

In addition, the internal standard approach allows the selection of genes with significantly higher variability compared with the majority of genes (stabile genes). As demonstrated previously (Dozmorov et al. 2004b; Dozmorov et al. 2005), the behavior of these hypervariable (HV) genes could tease out important clues about naturally occurring dynamic prosesses in the organism or experimental system under study.

The objective of this study was to develop and characterize an organotypic model of endometrial carcinogenesis and Flex-Het chemprevention and to identify molecules in this model that are regulated in opposite directions during the processes of carcinogenesis and chemoprevention. The poly-cyclic aromatic hydrocarbon and known carcinogen, 7,12-dimethylbenzanthracene (DMBA) was chosen to induce the cancerous phenotype, because it is well-characterized and has been shown to increase the incidence endometrial hyperplasia and uterine polyps in mice (Geary, 1984; Gray et al. 1997). Karyometric analysis and the soft-agar colony forming assay were used to confirm the development and prevention of the cancerous phenotype in the organotypic cultures (Ranger-Moore et al. 2005). Microarray analysis interpreted via the internal standard approach and identification of HV genes was used to characterize the dynamics of the carcinogenesis and chemoprevention mechanisms. In addition, multivariate procedures of correlative clustering and mosaic presentation (Dozmorov et al. 2003b) and modified discriminant function analysis (Dozmorov et al. 2005; Jarvis et al. 2004b) were used to identify carcinogen initiated changes in the functional associations of HV genes. Validation of differential expression of secreted proteins, tenascin C and inhibin A, that can contribute to driving the biological system modeled by our analysis was performed by Western blot and enzyme linked immunosorbant assay (ELISA).

## Materials and Methods

### Organotypic cultures

The cultures will be established, maintained and used to prepare organotypic cultures as previously described (Kamelle et al. 2002). The cultures used for this study were collected from a healthy pre-menopausal female who did not take hormones, dietary supplements or medications. Endometrial organotypic cultures were grown for 1 week to generate tissue histology prior to a 4 hour exposure to 5 μM DMBA to induce DNA damage or solvent control. After DMBA exposure, media were replenished, and 1 μM SHetA2 was added to half of the DMBA-treated cultures to prevent transformation and half of the solvent-treated cultures as a control for the effects of the drug alone. Media and SHetA2 were replenished every Monday, Wednesday and Friday for two weeks. One set of cultures from each experiment was fixed in formalin, embedded, sectioned (5 microns), deparaffinized and stained with hematoxylin and eosin (H&E). Parallel cultures were treated with collagenase for 15 minutes at 37 °C to release the cells, followed by addition of Phosphate Buffered Saline (PBS) and centrifugation to pellet the cells for RNA and protein isolation.

### Tissue preparation and karyometric feature extraction

Formalin-fixed paraffin-embedded blocks of the cultures were sent to an independent laboratory at the Arizona Cancer Center where they were sectioned, stained with H&E and evaluated microscopically for karyometric analysis as previously described (Ranger-Moore et al. 2005). Five micron tissue sections were scanned and the nuclei segmented from cellular background using custom software. One hundred nuclei were randomly selected from each sample for analysis. Ninety-five karyometric features that capture chromatin information at varying levels of complexity were measured. Most of these features fall into one of three broad categories, each of which summarizes information at increasingly higher dimensions of order. Zero-order statistics produce scalar features based on the values of all pixels in the nucleus, such as mean optical density (OD), OD variance, and nuclear area (total number of pixels). First-order statistics consist of vectors of features derived directly from pixel characteristics, such as the frequency histogram of OD values. Second-order statistics consist of matrices that generate features containing information on relationships between pixels, such as the similarity or difference of adjacent pixels on OD value, or pixel run lengths occurring at given OD levels. The full set of features captures a broad range of information that maximizes the opportunity to detect nuclear changes of significance in deviation from normal or a reference standard.

### Karyometric statistical analysis

The nuclear signature was obtained by first calculating the mean and standard deviation of karyometric features in normal endometrial cultures. The value of each feature in the treated cultures was standardized relative to the untreated controls. Thus, the standardized measure for each feature in each nucleus in the treated cultures indicates how many untreated-control standard deviations away from the untreated-control it lies. The absolute value of the distance is routinely used because the magnitude of the deviation is of concern. The nuclear signature was graphed as a bar chart of the 95 karyometric features, with the vertical height of each bar depicting the deviation from normal averaged across all nuclei. In normal tissue, the height of each bar was approximately 0.67, which is the typical absolute deviation in a normal distribution. Organotypic cultures treated with DMBA typically exhibited marked elevations in numerous karyometric features. A discriminant function (DF) was developed based on those karyometric features best able to distinguish between untreated and DMBA-treated cultures. To choose the appropriate karyometric features without imposing distributional assumptions, a non-parametric rank-sum test was used with a low significance level of 0.005 in light of the multiple comparisons being conducted. The 5 karyometric features showing the strongest differences between the two tissue types were then submitted to a DF analysis and the resulting function was applied to all samples from the study. These 5 features were total optical density, nuclear area, grey level nonuniformity (akin to, but not the same as, the standard deviation of optical density), run percentage (i.e. the percentage of pixels involved in homogeneous run lengths), and the total number of ‘grey’ pixels (i.e. pixels falling into an intermediate range of O.D.).

### RNA isolation and microarray analysis

RNA was isolated from frozen cell pellets using the mini RNeasy kit (Qiagen, Valencia CA) in combination with the Qiagen shredder. After purification, RNA concentration was determined with a Nanodrop scanning spectrophotometer (Nanodrop Technologies, Wilmington, DE), and then qualitatively assessed for degradation using the ratio of 28:18s rRNA using a capillary gel electrophoresis system (Agilent 2100 Bioanalyzer, Agilent Technologies, Santa Clara, CA). cDNA synthesis, hybridization and staining were performed as specified by Affymetrix (Santa Clara, CA). Briefly, 4.8 μg of total RNA was primed with T7-oligo-dT and reverse transcribed with SuperScript II, followed by production of double-stranded cDNA with *E coli* DNA polymerase. cRNA was transcribed *in vitro* from the T7 promoter using a biotinylated ribonucleotide analog and then fragmented to approximately 100 nt. cRNA is hybridized to human Affymetrix U133 Plus 2.0 GeneChips^™^ microarrays. These arrays contain approximately 47,000 probes for transcripts in the human genome. GeneChips^™^ were washed and stained using an Affymetrix automated fluidics station 450 and scanned with an Affymetrix 3000 7G scanner.

### Statistical analysis of differentially expressed genes

Our methods of data normalization and analysis are based on the use of internal standards that characterize some aspect of system behavior such as technical variability are used thus enabling an increase in statistical power. In general, an internal standard is constructed by identifying a large family of similarly behaving genes. For example, genes expressed below technical sensitivity comprise a background cohort while genes having a similar expression pattern across several distinct experimental conditions are denoted as an equally expressed cohort. These internal standards are used to robustly estimate parameters that describe some state of the experimental system such as the identification of genes expressed distinctly from background, differentially expressed genes, genes with differences in the expression variability and genes having similar dynamical behavior. The advantage of the methods based on the internal standard comparisons over traditional paired T test and ANOVA analysis is in their capability to increase statistical power of analysis (sensitivity) without loosing specificity of statistical criteria. This advantage results from the high representativity of the internal standards consisting normally of thousands of members (Dozmorov et al. 2003a; Knowlton et al. 2004; Dozmorov et al. 2004a).

Conclusions about differences in gene expression were based on the use of associative analysis described in (Dozmorov, Centola, 2003). Additional restrictions were applied to focus attention on the especially prominent differences: a minimum averaged expression level at least 10 times above background, a minimum 2-fold difference in the mean expression values between groups, and a minimum of 80% reproducibility using the jack-knife method—the leave-one out-cross validation—used to cross-validate results of differential analysis.

### Identificaton of hypervariable (HV)-genes

A ‘reference group’ was defined as genes in the untreated control cultures that were expressed above background with inherently low variability as determined by an F-test. Hyper-variable (HV) genes were identified as genes with expression levels that varied significantly (P < 1/N) in comparison to the ‘reference group’ (Dozmorov et al. 2004b). The threshold of P < 1/N, where N represents the number of genes expressed above background, is a slight modification of the Bonferroni correction for multiple hypothesis tests. This threshold usually identifies more than half of all genes on an array. It is important to note that such hyper-variable genes exist even within homogenous groups of samples such as the control group. Their variability statistically exceeds the established biologically stabile genes (BSG) variability and therefore may reflect some non-synchronized gene dynamics. Hyper-variations appearing from experimental errors (influence of dirty spots etc) were filtered from this analysis statistically by comparing the variability of the residuals in replicated group of samples with the same variability obtained after excluding both the maximum and minimum, one at a time. A statistical decrease in variability after excluding one replicate provides evidence of possible error in that particular replicate. Such genes were excluded from the family of HV-genes as being falsely selected. Once filtered, the expression patterns can be considered snapshots of some dynamical biological process in which they participate. Correlation of these expression patterns may reflect some functional interconnections in the aforementioned dynamical processes.

### Comparative cluster analysis of HV-genes

Genes selected as HV-genes in all three treatment groups (DMBA, DMBA+SHetA2, SHetA2) were clustered based on correlation of their expression. The clustering procedure was based on the Pearson correlation described in detail in (Dozmorov et al. 2003b) and consisted of the following steps: 1) Connectivity was defined for each gene as the number of other genes whose expression behavior in samples of the group correlated with a given gene—the appropriate threshold for the correlation coefficient used to define connectivity was calculated using a simulation study; 2) HV-genes were sorted by their connectivity and the clustering process was started with genes of higher connectivity to seed clusters. The gene of higher connectivity and all genes correlated with it comprised cluster 1. The next gene of the highest connectivity not belonging to cluster 1 was used as a seed for the cluster 2 and so on. The final result of this clustering is presented in the form of correlative mosaics in which gene relationships through correlation coefficients are expressed in colors.

### Promoter analysis

The strategy for analyzing the significance of gene selections with reversible dynamics was based on the assumption that co-regulated genes are likely to share common regulatory motifs (Chiang et al. 2001). Transcription elements over-represented within selected genes were determined with program PAINT 3.3 (Vadigepalli et al. 2003) available online (PAINT:), to query the transcription factor database (TRANSFAC) (BIOBASE:). PAINT 3.3 was employed to examine 2000 base pairs of regulatory regions upstream of the transcriptional start site of each differentially expressed gene from the microarray expression data. GeneBank accession numbers were used as the gene identifiers in PAINT input files. The probability of the observed pattern was determined by reference against the total genome. Results of this analysis are presented in heat map matrix with individual elements colored by the significance of the p-values: over-representation in the matrix is indicated in red (indicate p < 0.05).

### Correlation mosaics

Correlations coefficients and connectivity parameters for gene expression patterns were derived and used to generate Correlation Mosaics as previously described (Dozmorov et al. 2003c). Briefly, correlations between the expression patterns of genes were determined by first running simulation experiments to determine a background threshold level that the correlation needed to be above in order to be considered significant. Correlation coefficients were derived in a comprehensive pairwise manner. A connectivity parameter for each gene was then calculated from the number of other genes that exhibit a correlated expression pattern and was used to determine clustering of genes into a matrix.

### Validation of differential protein expression with Western blot and ELISA

At the end of the treatment period of the endometrial organotypic cultures, the media was removed and replaced with serum free media. The conditioned media was collected after 6 hours and concentrated with an Amicon protein concentrator. For tenascin-c analysis, 25 μg of protein concentrated media from each of the 4 treatments was loaded onto a 10% SDS gel and transferred to a PVDF membrane. Membranes were blocked with 5% milk and then incubated with an anti-tenascin C primary antibody (Abcam). The membranes were rinsed with Tris/Tween buffer and incubated with horseradish peroxidase (HRP) conjugated secondary antibody (Santa Cruz). After further rinsing, Luminol reagent (Santa Cruz) was used to detect specifically bound antibody. The bands were visualized by exposure to X-ray Film. For thymidine phosphorylase analysis, a mouse anti-thymidine phosphorylase antibody (Santa Cruz) was used to immunoprecipitate the protein from 1.5 ml of conditioned media before loading the SDS gel and the blot was hybridized with a goat anti-thymidine phosphorylase antibody (R&D Systems). For the Enzyme Linked Immunosorbant Assay (ELISA), 25 μl of 50X concentrated media from each treatment group was applied to duplicate wells of antibody coated microtiter strips from the inhibin-A ELISA kit (Diagnostic Systems Laboratories) and the ELISA was performed according to manufacturers instructions. This assay captures the dimer of inhibinβA/inhibinα called Inhibin A with an anti-inhibinβA antibody and recognizes the Inhibin A bound with a secondary anti-inhibinα antibody conjugated with horse radish peroxidase. Signal corresponding to the amount of bound protein was generated with a tetramethylbenzidine (TMB) substrate that was metabolized by the horse radish peroxidase to a color that was read the optical density (OD) of 450 nm with correction at 650 nm. The concentration of inhibin A in each well was determined by comparing the average optical density of duplicate wells of conditioned media with a standard curve generated from duplicate wells concentration standards in each of the two experiments.

### Discriminant function analysis for Microarray data

Discriminant function analysis (DFA) (Statistica, StatSoft, Tulsa, OK) was used for selecting a set of genes with maximal discriminatory capabilities between untreated controls and cultures receiving the treatments. A variant of DFA named the “Forward Stepwise Analysis” that has been successfully applied to clinical classification (Jarvis et al. 2004b; Jarvis et al. 2004a) and experimental microarray data (Dozmorov et al. 2003c) was used. In this modification of the DFA, only HV-genes were used for selection of discriminatory parameters, defined as roots. DFA based on the use of dynamical parameters (DDFA) is a classification method that uses differences in gene functional associations as a discriminatory factors (Dozmorov et al. 2005). The analysis was carried out using data normalized and adjusted with procedures described above. HV-genes with highest discriminatory capability were used for calculation of linear combinations (roots). These roots demonstrate relative stability for samples within groups and at the same time significant difference for samples from different groups. The use of these roots as coordinated gives visual presentation of the discriminatory rules obtained for the different treatment groups.

## Results

### Biological model

**Figure 1:**
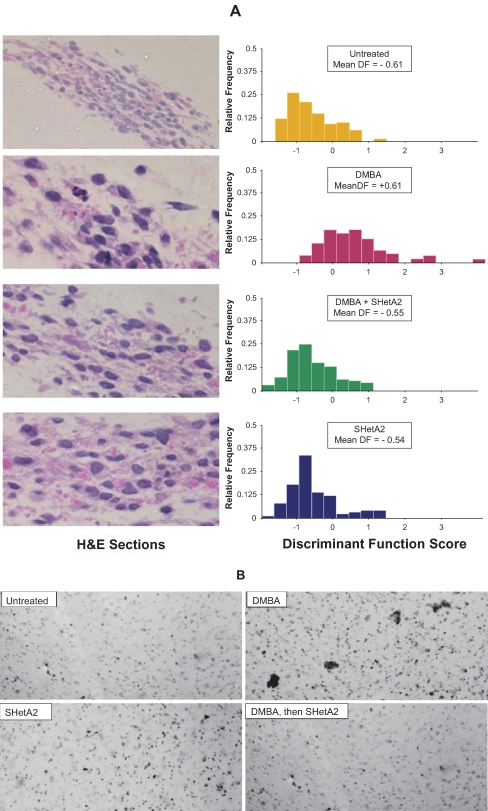
**A) Organotypic Cultures and Karyometric Analysis.** The panel on the left depicts photomicrographs of H&E stained sections of organotypic cultures that were fixed after the indicated treatments. The graphs on the right represent the profile of discriminant function scores for each of the treatments. **B) Clonogenic soft agar assay.** The ECC model was grown and treated as described in Figure 1. At the end of the treatment period, the collagen was digested and the cells released were pelleted and counted. Equal numbers of cells from each treatment were plated in soft agar in triplicate wells without DMBA or SHetA2. Fresh media without DMBA or SHetA2 was added twice per week to keep the cultures hydrated and nourished. After two weeks, cells were stained with crystal violet and photographed.

Endometrial organotypic cultures were exposed to DMBA or solvent control and then cultured in the absence or presence of SHetA2 for two weeks prior to fixation and evaluation for histological features. In greater than 6 repeats of this treatment schedule, consistent histological transformation to the cancerous phenotype in DMBA treated cultures and prevention of the cancerous phenotype in DMBA + SHetA2 treated cultures was observed (Fig. 1A).

This transformation and chemoprevention was validated by karyometric analysis at an independent laboratory at the Arizona Cancer Center. The value of each feature in the treatment groups was standardized relative to the untreated control. Three key karyometric features of the nuclei (total optical density, standard deviation of optical density, and nuclear area) showed dramatic and statistically significant increases in the cells in DMBA-treated cultures relative to untreated cultures. These three features and others were nearly identical in the cultures treated with DMBA + SHetA2 and control cultures treated with solvent only, consistent with SHetA2 chemoprevention activity. Treatment with SHetA2 alone significantly increased only one feature in comparison to untreated cultures—the standard deviation of the optical density. A linear discriminant function utilizing 5 texture features was developed to distinguish between untransformed and DMBA-transformed nuclei. The resulting vector of feature weights was then applied to the karyometric data for nuclei from cells treated with both DMBA and SHet2A or with SHetA2 only. The results of the karyometric analysis demonstrate that DMBA increases the numerical value of the discriminant function scores causing a shift to the right, and that this shift is prevented by SHetA2 (Fig. 1A). Treatment with SHetA2 only did not significantly affect the profile.

The ability of cells to form anchorage-independent colonies in soft agar is often used as a measure of transformation. Cells released from the organotypic culture at the end of the treatment period were plated into soft agar and their ability to form anchorage-independent colonies was monitored over 2 weeks. Consistent with the pathology review and karyometric analysis, only cultures treated with DMBA and cultured in the absence of SHetA2 exhibited the capacity to form colonies when plated in soft agar (Fig. 1B).

### Microarray analysis identifies genes associated with carcinogenesis and chemoprevention

**Figure 2:**
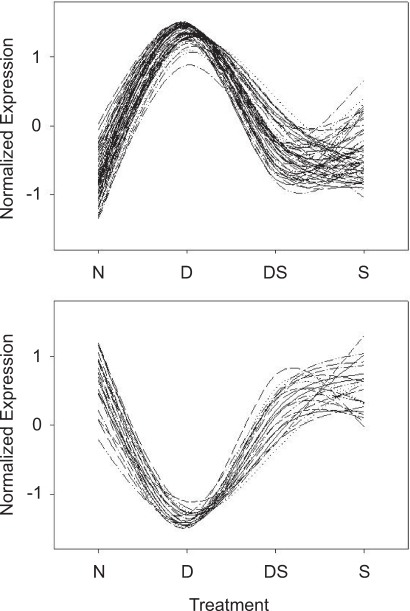
Reversible Dynamics. After the hypervariable genes in each treatment group were identified, genes were prioritized for further study if they exhibited all of the following: Significantly altered by DMBA (D) in comparison to the untreated control (N), Alteration observed in DMBA treated cultures was not observed in cultures treated with SHetA2 + DMBA (DS) or SHetA2 (S) alone. The rationale for this prioritization is that it will identify genes directly involved in the transformation process and not altered by other effects of DMBA or SHetA2. Eightynine genes were identified to fit these prioritization criteria. Normalized expression (y′) was derived from the following formula: y′ = (y−A)/SD, where y = the individual gene expression level, A = the gene expression averaged over all samples, and SD = standard deviation.

**Figure 3:**
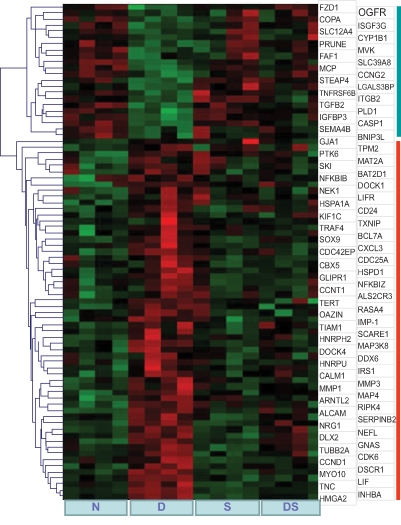
Hierarchical clustering of genes with “forward-back” dynamics. The right side colored bar presents two different clusters joined genes increased (red bar) or decreased (green bar) their expression after D action. Samples grouping is indicated in the bottom of the graph.

**Figure 4:**
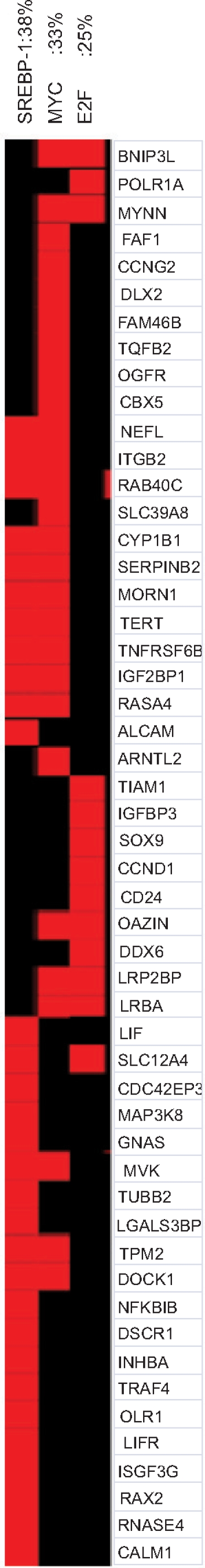
Promoter analysis of genes with “forward-back” dynamics. Promoters are shown along abscissa (on the top) and genes along ordinate. Red spots indicated genes with over represented promoters relatively total genome (statistical threshold is p = 0.05).

**Figure 5:**
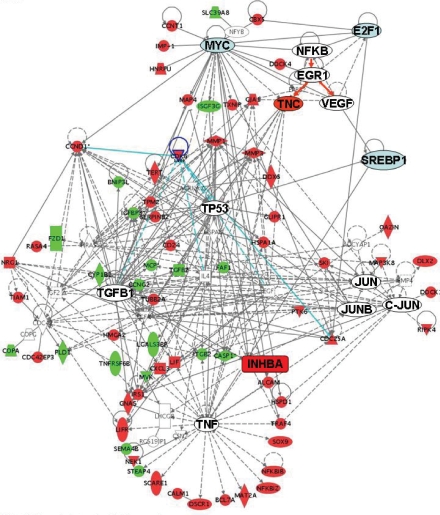
Ingenuity pathway analysis of gene demonstrating “reversible” dynamics. Red symbols—genes with significant increase of expression after D action, green symbols—genes decreasing expression after D action. The genes validated as shown in Figure 8 to be differentially expressed at the protein level, tenascin-C (TNC) and inhibin A (INHBA), are represented as larger symbols for ease of recognition. Blue symbols—the main transcription factors involved in regulation of the differentially expressed genes (by results of promoter analysis—see Fig. 4. Solid lines—direct interactions, dashed—indirect. Node symbols: squares—cytokines, rombs—enzymes, cyrcles—all others. Not colored symbols indicate genes added by the program for completeness of connectivities.

RNA isolated from 4 replicate cultures grown in parallel with those used for the karyometric validation was evaluated with microarray analysis to identify candidate genes and pathways involved in the carcinogenesis and chemoprevention processes. Paired comparisons of each of the treatment groups with the untreated control group identified several hundred genes that were significantly altered in expression by each treatment. The strategy to identify a gene subset most likely to be directly involved in the carcinogenesis and chemoprevention processes was to select genes altered by DMBA, reversed by SHetA2 after DMBA treatment, and not affected by SHetA2 only treatment. The rationale for this strategy is that carcinogenesis false-positives in the DMBA-only treated cultures can be reduced by selecting only the subset of these genes that were counter-regulated by SHetA2 chemoprevention in the cultures treated with DMBA followed by SHetA2. Also, chemoprevention false positives in the SHetA2-only treated cultures were reduced by eliminating those genes that were not significantly altered in the SHetA2-only versus DMBA followed by SHetA2 treated cultures. A refined list of 89 genes that were counter-regulated by DMBA and SHetA2 were identified. The two patterns of expression demonstrating the reversible dynamics of these genes are depicted in Fig. 2. Hierarchical clustering of these genes is shown in Figure 3. Analysis of the 89 gene promoters (Fig. 4) identified leading roles of several transcription factors in the observed changes of gene expression. Evaluation of the signaling pathways directly influenced by these 89 prioritized genes was performed using Ingenuity software (Fig. 5). Several oncogenic networks were found to be directly regulated at multiple points, the most prominent of which were c-Myc, p53, and tumor necrosis factor-α (TNFα) and genes of Activator Protein-1 (AP-1) family (JUN, JUNB, C-JUN). Three of the transcription factors (SRE-BF1, MYC (c-Myc), and E2-F1) in the promoter analysis were also identified by Ingenuity Pathway software as being incorporated into the network of selected genes (Fig. 5).

### Hypervariable gene analysis to identify differences in functional associations

A systems biology approach was use to identify networks of genes that responded to DMBA and SHetA2 in association with each other suggesting functional interaction. The first step in this approach was to identify hypervariable (HV) genes. HV genes are defined as genes that demonstrate excessive variation that can not be explained just as normal fluctuations of expression. We hypothesized that this variability is a reflection of dynamic processes that the HV-gene encoded proteins are involved in. These processes are not synchronized in the samples, even in a very homogenous group. This means that the processes are in different phases of the same process in different samples. Additional evidence for this integrative dynamical process is provided by the fact that many of HV-genes are changing together, i.e. they have highly correlated expression profiles within groups and sometimes also between groups (Dozmorov et al. 2005; Dozmorov et al. 2004b). HV-genes were selected in all 4 groups of samples as having variability significantly exceeding experimental noise variability and that appeared hypervariable in at least 3 treatment groups were selected for further study. After exclusion of all duplicates and functionally non-characterized genes, there were 61 genes remaining that matched these criteria. Several genes that have previously described associations with carcinogenesis are presented in Table 1.

### Functional associations between the 61 HV-genes

**Figure 6:**
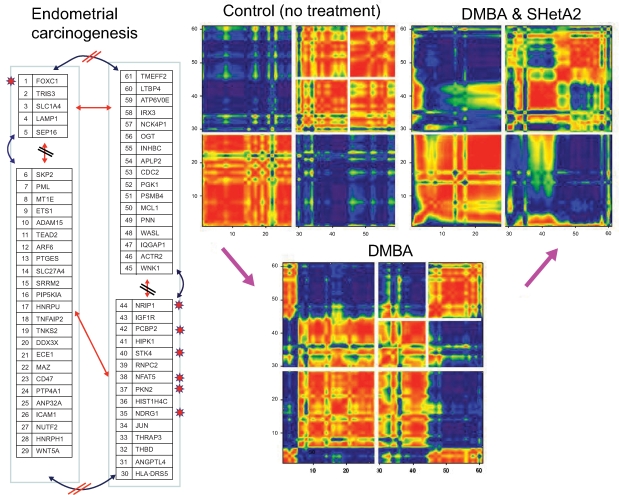
Correlative mosaics of HV-genes in normal control group, after DMBA treatment, and after combined DMBA and SHetA2 treatment. The orders along the axes were chosen to group genes belonging to the biggest clusters in the normal control. The same order is kept in all other mosaics. The colors of spots in the mosaic characterize correlation between genes used as coordinates for given spot: red—positive correlation, blue—negative. The lists of HV-genes are presented in blocks consistant with their cluster allocations. Changes in cluster associations in course of carcinogenesis are presented here with arrow connections. The genes used for the DFA-analysis in Figure 8 are marked here with stars.

Correlation mosaics were generated to visualize how the expression of HV genes are related to each other and to compare how these relationships are altered by the different treatments. Correlation coefficients for the expression patterns of genes were derived in a pairwise fashion and used to determine the number of other genes that significantly correlated with each gene (connectivity index). The connectivity index for each gene was used to determine the order it was represented along an axis of a matrix. The first cluster of genes was seeded with the gene that had the highest connectivity index. All of the genes that had significant correlation coefficients were represented alongside. The second cluster was seeded with the gene that was not included in cluster 1 and had the highest connectivity index. Additional clusters were generated until all of the genes were used up. The visual presentation of this clustering of genes into a matrix is a correlation mosaic (Fig. 6) where coordinates represent the numbers of a given gene in the list (as shown in Fig. 6) and the color of a given spot indicates the type of correlation between the genes represented by its coordinates. Red color is used for positive correlation and blue for negatives correlation.

**Figure 7:**
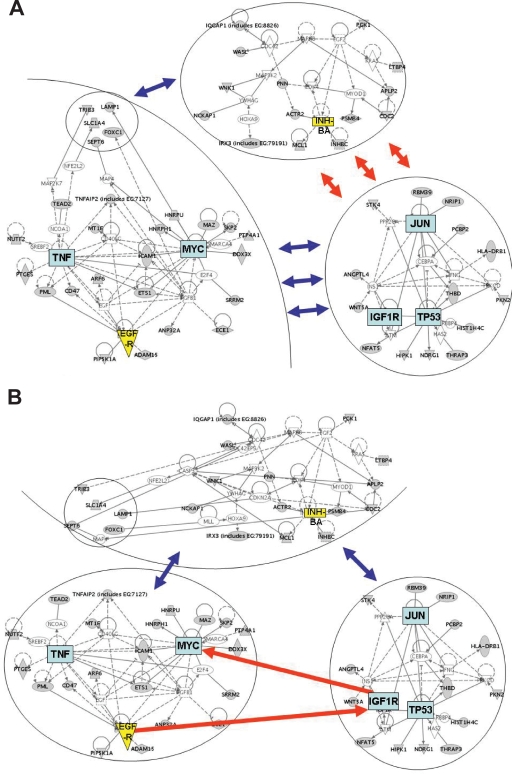
Ingenuity pathway analysis for group of HV-genes demonstrating reversible changes in cluster associations as it is shown in Figure 6. The red arrows indicate tight associations and the blue arrows indicate strong negative associations or repression. Panel **A** represents associations for the normal control group, and Panel **B**—for DMBA treated group.

Genes used in this clustering procedure also are shown in their functional associations in Figure 7A (no treatment) and Fig. 7B (DMBA treatment). This mosaic presentation demonstrates the presence of three large clusters linked with tense correlative associations: cluster A—genes 1–29, cluster B—genes 30–44, and cluster C—genes 45–61. These clusters are also presented as tightly interconnected associations in Figure 7A and B. The main changes in mosaics created for DMBA carcinogenesis are in the associations between these clusters. In the normal control group, cluster B merges with cluster C in one big cluster that is in antagonistic association with cluster A. In the DMBA treated group, cluster B merges with cluster A and this new big cluster is mainly in negative association with cluster C, except for a small fraction of the cluster A (first five genes), that change their preference from A to C. In the DMBA+SHetA2- treated group these associations are partially reconstituted to their normal control state.

### Validation of microarray and HV Gene functional interaction results

**Figure 8:**
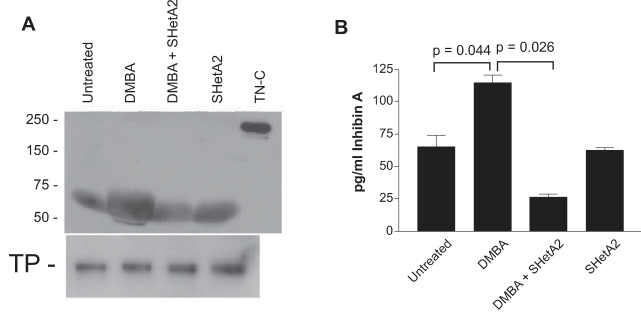
Tenascin C and Inhibin A secretion in ECC model. Endometrial organotypic cultures were exposed to 5 μM DMBA for 4 hours followed by two weeks of growth −/+ 1 μM SHetA2. At the end of the experiment, cultures were switched to serum free media, the conditioned media collected after 6 hours. **A)** Western blots of conditioned media. The top blot was hybridized with anti-tenascin C and the bottom with anti-thymidine phosphorylase as a control for a secreted protein that is not significantly regulated in the ECC model. **B)** ELISA of Inhibin A levels in the conditioned media. Error bars represent standard error. The two p values represented are from two tailed paired t-tests. There were no significant differences between other pairwise comparisons. Data shown for each protein are from the same experiment, and are representative of two independent experiments performed in quadruplicate.

As a step toward validation of this model, two genes, tenascin c (TN-C) and inhibinβA (INHBA), demonstrating reversible expression from Figure 2 that were subsequently identified as playing functional roles in the ECC model by Ingenuity Analysis (Fig. 5) and by cluster analysis (Fig. 7) were evaluated in the organotypic model. Western blot analysis of conditioned media from organotypic cultures confirmed that TN-C secretion followed the pattern of being induced by DMBA transformation, repressed by SHetA2 chemoprevention and unaffected by SHetA2 treatment alone (Fig. 8A). Since there are no standard secreted proteins that can be used as a control for protein loading in conditioned media, we chose to use another secreted glycoprotein, thymidine phosphorylase (TP also called platelet derived endothelial cell growth factor or PD-ECGF), which provides a demonstration of a protein that does not exhibit altered levels in this model. Interestingly, TN-C was fragmented in serum-free media but remained intact in the presence of serum as observed by others (Wallner et al. 2004; Jones et al. 2002). The 65kD fragment also was observed in human carotid endarterectomy specimens and found to retain the EGF-like domain and induced apoptosis, while intact TN-C did not induce apoptosis (Wallner et al. 2004; Jones et al. 2002).

ELISA analysis of inhibin A, which consists of an inhibinβA/inhibinα demonstrated a consistent pattern of expression with that observed in the microarray analysis with increased levels in the DMBA transformed cultures, decreased levels in the SHetA2 chemoprevented cultures, and insig-nificant alterations in the cultures treated with SHetA2 only (Fig. 8B).

### Discriminant function analysis of microarray data

**Figure 9:**
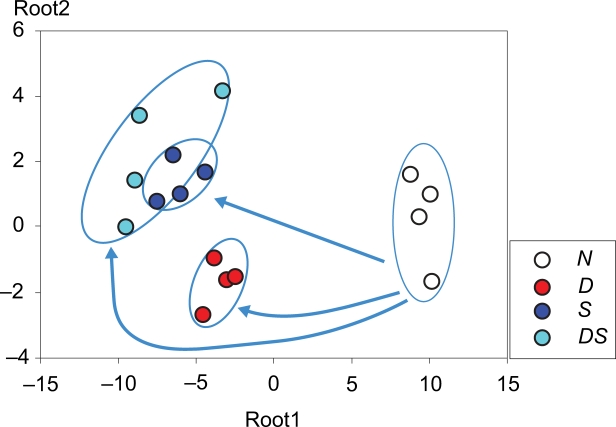
Discriminant Function Analysis of all group of samples. All HV-genes presented in mosaics Fig. 6 were used for this analysis. As a result of analysis there were selected 7 of them (Table 2) with higher discriminatory capability for calculation of linear combinations – ROOTS used as coordinates: for presentation of the results of discrimination. Open circles present samples before stimulation and filled circles – after treatment.

Discriminant Function Analysis (DFA) is a statistical method to identify parameters that can distinquish between groups. It also can be used to identify which group an individual sample belongs to and ultimately could be used as diagnostic and prognostic assays for cancer. Discrimination of groups of samples is usually based on sets of differentially expressed genes that provide the best discrimination between groups. We developed an original modification of this method named Dynamical DFA that is based on the use of HV genes for discrimination. (see details in (Dozmorov et al. 2005)). All these genes are not differentially expressed between groups, and when considered separately from others, are not discriminatory, but their linear combinations created with statistical algorithms could be very discriminative and used for classification. All of the HV-genes presented in Figure 6 were used in Dynamical DFA analysis. The program selected 7 HV-genes (marked with stars in Fig. 6) for creation of discriminatory parameters called ROOTS, which are linear combinations of these 7 genes with appropriate constant coefficients. The results of Dynamical DFA are presented in Figure 9. In fact, satisfactory group separation (only slightly overlapping) can be achieved with use of even smaller numbers of genes—only the first three in the list (Table 2).

## Discussion

In this study, karyometric analysis validated the consistent development and prevention of a transformed phenotype in endometrial organotypic cultures exposed to DMBA followed by treatment with solvent only or SHetA2, respectively. Microarray analysis identified genes that were differentially regulated during the processes of carcinogenesis and chemoprevention. Evaluation of the functional interactions of these differentially regulated molecules identified the involvement of several interacting signaling pathways regulated by TNFα, c-Myc, p53, and multiple Jun genes. Multiple members of these signaling pathways were directly altered, while these key regulators themselves were not (Fig. 5), suggesting that the functions of the protein products, not their mRNA expression levels, are involved in the mechanisms driving carcinogenesis and chemoprevention in this model. Each of these proteins function to directly or indirectly regulate gene expression, which could account for their functional involvment in the differentially regulated gene expression patterns observed. The activator protein-1 (AP-1) transcription factors (Jun and Fos families of proteins), TNFα, c-Myc and p53 are well-recognized for playing key roles in carcinogenesis and chemoprevention. Their specific association with DMBA-induced carcinogenesis and chemoprevention supports the validity of this model.

Evidence for direct involvement of TNFα in DMBA carcinogenesis is provided by the observations that TNFα deficient mice are resistant to DMBA induced carcinogenesis (Suganuma et al. 1999; Moore et al. 1999) and that neutralization of TNFα with a specific anti-TNFα antibody decreased tumor formation (Scott et al. 2003). Down-regulation of TNFα is involved in the mechanism by which several natural and synthetic compounds prevent DMBA carcinogenesis of the endometrium (Onogi et al. 2006; Tagami et al. 2004; Lian et al. 2002) and skin (Komori et al. 1993; Haranaka et al. 1988; Zhaorigetu et al. 2003).

The AP-1 signal transduction pathway is strongly implicated as a down-stream mediator of TNFα-driven carcinogenesis. AP-1 responsive genes are differentially regulated in TNFα-wild type (DMBA-sensitive) mice in comparison to TNFα-knockout (DMBA resistant) mice (Arnott et al. 2002; Brenner et al. 1989; Hartl et al. 2006; Chen et al. 2004). The AP-1 DNA binding site is requried for TNFα-induction of a molecule strongly implicated in carcinogenesis, intercellular adhesion molecule-1 (ICAM-1) (Chen et al. 2004). Increased expression of c-Jun and other members of the AP-1 transcription factor family is also associated with prevention of DMBA-driven endometrial carcinogenesis (Onogi et al. 2006; Tagami et al. 2004; Lian et al. 2002).

AP-1 activation could lead to the induction of c-Myc in DMBA carcinogenesis through a specific AP-1 DNA binding site in the c-Myc promoter known to be activated by AP-1 (Iavarone et al. 2003). Both AP-1 and c-Myc could then lead to induction of p53 through specific DNA binding sites in the p53 promoter that are known to drive synergistic increase in p53 when bound by AP-1 and c-Myc transcription factors (Kirch et al. 1999). Upregulation of the p53 tumor suppressor and down regulation of c-Myc protein function is associated with prevention of DMBA-carcinogenesis in several animal models (Todorova et al. 2006; Arora et al. 2004). The complex role of c-Myc in promoting growth and inducing apoptosis could explain why down- or up-regulation of c-Myc contributes to carcinogenesis depending on the cellular context (Vita et al. 2006).

TNFα, c-Myc, p53 and the AP-1 transcription factors are also known to be altered in human endometrial cancer specimens. High levels of TNFα were observed in serum (Diab et al. 2006a) and genotypic changes in the TNFα gene promoter were observed in tumor samples (Sasaki et al. 2000) from endometrial cancer patients. The c-myc gene is frequently amplified in endometrial cancer specimens (O’Toole et al. 2006) with increased frequency in advanced, as compared to early endometrical cancers (Konopka et al. 2004). Increased c-Myc expression is associated with progression from the pre-cancerous endometrial atypical hyperplasia to invasive cancer (Dong et al. 2004b) and with development of lymph node metastases (Dong et al. 2004a). Mutation and altered expression of p53 occurs frequently in human endometrial cancer (Jeon et al. 2004) and is associated with high serum levels of TNFα (Diab et al. 2006b). Increased levels of AP-1 transcription factors have been observed in endometrial cancers in association with regulators of cell cycle progression (Bamberger et al. 2001).

c-Myc was also implicated in the carcinogenesis and chemoprevention model through promoter analysis, which demonstrated that the majority of differerentially expressed genes are under positive control of c-Myc along with along with E2-F1 and SRE-BP1 (Figs. 5 and 6). These transcription factors could participate in carcinogenesis synergistically, because c-Myc increases induction of E2-F1 in mouse tumor cells (Liao et al. 2000; Benaud et al. 2001), and E2-F1 increases expression of c-Myc mouse liver carcinoma (Conner et al. 2000). SRE-BP1 appears to drive alterated expression of lipid metabolizing enzymes in a liver carcinogenesis model driven by c-Myc and E2-F1 (Coulouarn et al. 2006). Acting together and synergistically, these transcription factors could be responsible for most of the changes in gene expression patterns associated with DMBA initiated carcinogenesis.

Additional validation of this model is provided by the identification of Nuclear Factor-κB and Cyclin D1 (NF-κB and CCND1 in Fig. 5) as playing functional roles in the ECC model. In Figure 5, NF-κB is colored white, indicating that NF-κB is not directly regulated in expression but instead plays a functional role in the model, which is consistent with the observed SHetA2 inhibition of NF-κB activity, but not expression, in multiple cancer cell lines (Chengedza et al. 2007). CCND1 is red in Fig. 5, indicating that it is increased in DMBA-transformed cultures and decreased by SHetA2. This is consistent with the known increase of CCND1 in many cancers and the observed decrease of CCND1 in multiple cancer cell lines (Tashiro et al. 2007; Masamha, 2007). While these genes may not be directly targeted by DMBA, they appear to be involved in the down-stream events that occur during the two weeks of culturing subsequent to the DMBA exposure and appear to be involved in the SHetA2 chemoprevention mechanism.

HV-gene analysis identified a major switch associated with DMBA carcinogenesis that gathered together the TNFα pathway, major components of the AP-1 transcription factors and the c-Myc oncogene into a tightly associated team that appears to synergetically initiate endometrial carcinogenesis. These factors have a negative association in untransformed tissue and the SHetA2 treatment reconstituted the negative associations between these components, thus acting as a cancer preventing agent.

Cluster analysis of the HV-gene functional associations demonstrated that carcinogenesis in this model is accompanied by the appearance of the very positive (stimulating) association between a module of genes that includes TNFα, c-Myc and Epidermal Growth Factor Receptor (EGF-R) genes from one side and a module that includes p-53, Insulin-Like Growth Factor I Receptor (IGF-1R) and multiple Jun genes from another (Fig. 7B). In the normal state, and with the additional cancer preventing action of SHetA2, there is a strong negative association between these modules (Fig. 7A). EGF and IGF are potent survival factors, and their receptors are often constitutively activated in cancer cells leading to increased proliferation and resistance to apoptosis (Jones et al. 2005; Sekharam et al. 2003). The functions of their membrane receptors, EGF-R and IGF-1R, are integrated through a physical complex where these two receptors activate each other (Ahmad et al. 2004; Burgaud et al. 1996; Huether et al. 2006) Thus, the increased association of the interaction of these receptors in DMBA-treated cultures is likely to further enhance their induction of proliferation and resistance to apoptosis. In support of this, combined inhibition of EGF-R and IGF-1R by Gefitinib (Iressa) and AG1024, respectively, resulted in additive to synergistic inhibition of growth and induction of apoptosis in breast cancer cell lines (Camirand et al. 2005). The functional interaction of these proteins in cancer is further demonstrated by the known role that IGF-1R plays in development of resistance to EGF-R inhibitors (Jones et al. 2005; Steinbach et al. 2004; Chakravarti et al. 2002).

Increased interaction of IGF-1R with c-Myc and TNFα in the transformed cells can also drive carcinogenesis. The tumor cell microenvironment appears to govern the ability of IGF-1R to enhance or inhibit the pro-apoptotic activity of TNFα, (Remacle-Bonnet et al. 2005). Induction of c-Myc expression by IGF-1R activity can increase resistance to apoptosis in cancer cells (Sumantran et al. 1993; Grandori et al. 2000; Ceballos et al. 2000).

In an effort to provide evidence for the biological validity of the molecular mechanisms characterized by the bioinformatics approach, the expression of proteins encoded by genes that exhibited “reversible” expression and functional involvement in the modeled systems driving carcinogenesis and chemoprevention were evaluated. Secreted proteins were chosen for evaluation because they are most likely to be detected in blood and are therefore amenable to development of clinically applicable biomarker assays. The confirmation of the reversible expression (upregulated by DMBA transformation, reversed back to untreated levels by SHetA2 and unaffected by SHetA2-only treatment) of the secreted proteins, TN-C and INHBA, which were identified in the modeling, but not TP, which did not appear in the modeling, adds validity to the microarray analysis and provides additional insight into the biological system driving our model.

TN-C can activate EGF-R (Swindle et al. 2001) and therefore may be involved in the altered relationship-between EGF-R and IGF1R that occurs during carcinogenesis in our model (Fig. 7). The TN-C growth-promoting protein has EGF-like repeats that induce autophosphorylation of EGF-R leading to activation of the receptor and increased cell proliferation and adhesion (Swindle et al. 2001). The ability of IGF-1 to increase TN-C protein deposition (Kenney et al. 2003) and of TNFα to alter TN-C mRNA splicing (Latijnhouwers et al. 2000) adds further synergy and validity to this model. In clinical studies, TN-C expression is increased in preneoplastic lesions of the endometrium (Sedele et al. 2002) and in the serum of cancer patients (Sato et al. 2006). Serum levels of TN-C appear to be associated with tumor progression in pancreatic carcinogenesis (Esposito et al. 2006) and melanoma (Burchardt et al. 2003) and with angiogenesis and prognosis in non-small cell lung cancer (Ishiwata et al. 2005). Tissue levels of TN-C are particularly high at invasive borders of cancer and appear to be indicative of poor prognosis in multiple tumor types including glioma, breast, cervix and ovary (Chiquet-Ehrismann et al. 2003; Buyukbayram et al. 2002; Wilson et al. 1996; Ioachim et al. 2002; Jahkola et al. 1998; Ishihara et al. 1995). Some studies suggest that different splicing variants of TN-C may have distinct roles in tumor physiology, diagnosis and prognosis in multiple cancers including endometrial adenocarcinoma (Chiquet-Ehrismann et al. 2003; Vollmer et al. 1997). As a single biomarker, TN-C is likely to have poor specificity for endometrial cancer, because it is also increased in many additional diseases associated with tissue damage (Sato et al. 2006). It’s role in EGF and IGF-1 signaling and increased secretion in cancer however, indicate that it plays a significant role in endometrial carcinogenesis and therefore is a rationale target for development of drugs and biomarker panels.

The other secreted glycoprotein InhibinβA (INHBA) chosen for validation analysis is a member of the inhibin/activin branch of the Transforming Growth Factor β (TGFβ) family of extracellular signaling molecules. There is a single inhibin α protein and 5 inhibin β proteins (inhibin βA, B, C, D and E). The inhibin β proteins function as homodimers to form activin A (βA/βA), activin B (βB/βB) and activing AB (βA/βB) or as heterodimers with inhibin α to form inhibin A (α/βA) and inhibin B (α/βB). The activins activate and the inhibins inhibit the Smad signaling pathway through uncharacterized Type II and Type I receptors (Robertson et al. 2004). We chose to evaluate inhibin A expression because a strong co-localisation of inhibin α and inhibin βA was observed in malignant endometrial tissue, which suggests that these tumors might be producing Inhibin A (Mylonas et al. 2006). Similar to TN-C, inhibin A, is not likely to be sufficiently specific for endometrial cancer to be used as a single biomarker because it’s expression levels are altered by ovarian function (Welt, 2002), but it may be usefull as a component of a biomarker panel that takes into consideration it’s relationships with other biomarkers. In our modeling of HV gene functional interactions (Fig. 7), the INHBA component of inhibin A exhibited a reversible relationship with IGF-1R. The prominent roles of both IGF-1 and inhibin A in ovarian function (Welt, 2002) suggests that there may be an interaction between these two proteins that affects development of endometrial cancer. Further studies are planned to evaluate the roles and interactions of individual inhibin proteins in endometrial carcinogenesis and chemoprevention.

In conclusion, karyometric analysis supports the validity of an organotypic model of endometrial carcinogenesis and chemoprevention by confirming the development and prevention of the cancerous phenotype. A systems biology approach implicated the involvement of several key molecules and oncogenic pathways in the processes of carcinogenesis and chemoprevention in this model. Our challenges in targeting these molecules for drug development will be to understand their mechanistic roles in carcinogenesis and chemoprevention, while our challenge in targeting them for biomarker development will be to determine if they are expressed at detectably and significantly higher levels in serum of women with endometrial cancer in comparison to health controls and patients with other diseases.

## Figures and Tables

**Table 1:** HV-Genes from Figure 6 known to be associated with cancer.

# In Figure 6	Name	Description	Association with cancer
10	ADAM15	ADAM metallopeptidase domain 15 (metargidin)	Is significantly increased in multiple types of adenocarcinoma (Kuefer et al. 2006)
9	ETS1	v-ets erythroblastosis virus E26 oncogene homolog 1 (avian)	An oncogene that plays important roles in cell proliferation, differentiation, lymphoid cell development, transformation, angiogenesis and apoptosis (Pei et al. 2005)
12	ARF6	ADP-ribosylation factor 6	Plays a critical role in ERK activation and tumor cell invasion (Hoover et al. 2005)
37	PKN2	Protein kinase N2	Plays an essential role in oncogenic cell transformation (Zeng et al. 2003a)
40	STK4	Serine/threonine kinase 4	Is involved in a tumor suppressor pathway (O’Neill et al. 2005)
44	NRIP1	Nuclear receptor interacting protein 1	Directly targeted by estrogen receptor in breast tumor cells (Lin et al. 2004a)
45	WNK1	WNK lysine deficient protein kinase 1	Ser/Thr Kinase induced by osmostic stress in cancer cell lines (Lenertz et al. 2005)
47	IQGAP1	IQ motif containing GTPase activating protein 1	Its expression pattern changes in the course of cancer progression (Cai et al. 2005)
48	WASL	Wiskott-Aldrich syndrome-like	Key regulator of dynamic changes in the actin cytoskeleton and cell migration in cancer cells (Sturge et al. 2002)
49	PNN	Pinin, desmosome associated protein	Participates in carcinogenic cell transformation (Shi et al. 2000)
54	APLP2	Amyloid beta (A4) precursor-like protein 2	A novel cancer marker (Mauri et al. 2005)

**Table 2:** Discriminant functional analysis summary.

# In Figure 6	Name	Description	Wilk’s Lambda	p-level	Associations with cancer
45	NDRG1	N-myc downstream regulated	0.0093	0.021	Significant correlation between NDRG1 expression and the histologic grade of tumors (Fotovati et al. 2006)
30	NRIP1	Nuclear receptor interacting protein 1	0.0019	0.219	Response element in breast tumor cells (Lin et al. 2004b)
28	PCBP2	Poly(rC)-binding protein 2	0.0194	0.007	Stimulate the activity of the proto- oncogene c-myc (Evans et al. 2003)
25	CTK4	Serine/threonine kinase 4	0.0145	0.011	Involved in tumor suppressor pathway (O’Neill et al. 2005)
21	PKN2	Protein kinase C-like 2	0.0058	0.041	Essential role in oncogenic cell transformation (Zeng et al. 2003b)
37	FOXC1	Forkhead box C1	0.0106	0.017	Upregulated in synovial sarcomas (Lappohn et al. 1989)
22	NFAT5	Nuclear factor of activated T-cells 5, tonicity-resonsive	0.0049	0.054	Involved in promoting carcinoma invasion (Jauliac et al. 2002)

NDRG1—the most changed from norm in D group (Fig. 1, E)

NRIP1—highest reconstitution factor (Fig. 2)

PCBP2—highest changes from norm in D group (Fig. 1, E)

STK4—high reconstitution factor (Fig. 2)

PKN2—high reconstitution factor (Fig. 2)

FOXC1—deviation form nor characteristic for S (Fig. 1, F, G)

NFAT5—high reconstitution factor (Fig. 2)
